# Use of Netnography to Understand GoFundMe^®^ Crowdfunding Profiles Posted for Individuals and Families of Children with Osteogenesis Imperfecta

**DOI:** 10.3390/healthcare10081451

**Published:** 2022-08-02

**Authors:** Argerie Tsimicalis, Michael Gasse, Marilyn Morand, Frank Rauch

**Affiliations:** 1 Ingram School of Nursing, Faculty of Medicine and Health Sciences, McGill University, Montreal, QC H3A 2M7, Canada; michael.gasse@mail.mcgill.ca (M.G.); marilyn.morand@mail.mcgill.ca (M.M.); 2 Shriners Hospitals for Children, Montreal, QC H4A 0A9, Canada; frank.rauch@mcgill.ca; 3 Department of Pediatrics, Faculty of Medicine and Health Sciences, McGill University, Montreal, QC H4A 3J1, Canada

**Keywords:** netnography, healthcare costs, osteogenesis imperfecta, costs, costs of illness

## Abstract

Osteogenesis imperfecta (OI) is a rare genetic disorder associated with low bone density and increased bone fragility. OI can lead to a variety of supportive and medical care needs; yet financial impacts for families and individuals living with OI remain understudied and largely invisible. Efforts by families to recover costs through GoFundMe^®^, the most important crowdfunding web platform worldwide, offer an unprecedented opportunity to gain insight into OI costs. The purpose of this study was to describe GoFundMe^®^ profiles and determine what factors may contribute to funding goal achievement. A netnographic approach was used to investigate a publicly available dataset from GoFundMe^®^, with 1206 webpages extracted and 401 included for analysis. Most webpages originated from the United States and were created by family members. Nineteen cost categories were identified. Thirty-seven web profiles met their funding goal. Funding increases or goal achievements created for children were associated with increased social-media exposure (i.e., Facebook). This study helped to describe and showcase the financial impacts of OI and effectiveness of a crowdfunding website to alleviate costs. The results highlight the need for further research to better understand OI costs and provide economic supports for individuals with OI.

## 1. Introduction

Osteogenesis Imperfecta (OI) represents a rare heterogeneous group of heritable bone fragility disorders occurring in 1 in 15,000–20,000 births [1]. Disorders in this group are caused by variants in multiple different genes that may be autosomal dominant or recessive [2]. Regardless of the pathology, those affected with OI will invariably experience susceptibility to bone fractures along a spectrum of severity, ranging from a subtle increase in lifetime fracture frequency to a high frequency of fractures, including prenatal fractures [2]. Treatment of children with OI requires an interprofessional, evidence-informed approach with medical, surgical, and rehabilitation interventions, along with the provision of supportive care from allied health professional and community networks. Underlying this treatment is a costly element, consisting of increased utilization of healthcare and social services [3,4,5]; provision of family caregiving resulting in forgone leisure, employment and school [4,6]; varying out-of-pocket expenditures, including expensive adaptive equipment to safely function at home and in society [3,4,5]; and varying psychosocial costs [4,6,7,8,9].

Preliminary evidence derived from seven countries suggests these costs associated with OI may be substantial for families [10], similar to other families caring for chronically ill or disabled children [9,10,11]. Families caring for a child with OI may encounter financial distress when confronted with costs which may be ineligible for private or public insurance funding [4,10,12], potentially leading to poverty. For example, among 25 Polish families studied, 48% were of low affluence [6]. Meanwhile, nearly one-third (29.6%) of the 34 participating families reported financial burden, defined as spending over 10% of their net income on OI expenditures, despite being cared for within the universal healthcare system of Canada [13]. The frequency of medical appointments, unpredictable nature of fractures, along with few daycares safely adapted for children with OI may lead to the need for a “stay-at-home” parent, which further compounds productivity losses and forgone income [4,9,12]. These financial difficulties may contribute to stress and compromise parents’ abilities to cope with stress [14].

Efforts to recover the costs associated with an illness have resulted in a global movement of individuals turning to crowdfunding websites [1,15]. These webpages may be an indication that individuals with a chronic illness, including families of children with OI, may be bearing a catastrophic cost [16]. GoFundMe^®^ is the most popular crowdfunding website in the world [https://www.gofundme.com, Last accessed on 1 May 2022], with 92.4% of its appearance in search engines being organic (i.e., retrieved due to relevance to search terms) as opposed to a paid advertisement [17]. GoFundMe^®^ also has a large social media presence, with 53% of traffic originating from social media, primarily Facebook, Inc., another popular website [17]. The data derived from GoFundMe^®^ offers a unique insight into costs associated with OI, but an innovative approach is required for analysis of publicly available, internet-based data. Netnography, a research methodology adapted to the study of online social networks [18], aligns with the innovative approach needed for analysis of public internet data. Netnography was first used by Dr. Robert Kozinets to study online fan discussions concerning the Star Trek franchise [18]. Netnography differs from ethnography by targeting online communities, focusing on multimedia communication and making use of readily available secondary digital data [18], whereas ethnography is a branch of anthropology interested in studying social groups from a participative–observational perspective [19]. Netnography is perceived as less intrusive than ethnography due to the lack of physical researcher presence, and has the potential for retroactive use [18]. According to Kozinets [18], any approach can be classified as netnographic, provided the research aligns with the following criteria: (1) participant observation; (2) an attempt to describe the human element of online human interaction and technological interaction, social interaction, and experience; (3) a focus on primarily online data; (4) adherence to strict and widely accepted standards of ethical online research; and (5) human intelligence and insight as a major part of data analysis [18]. Netnography has had recent applications in healthcare, including 64 citations in PubMed as of 20 February 2022, and has improved the fields of orthodontics [20], obstetrics [21], and the domain of online breast cancer support [22].

## 2. Materials and Methods

Following institutional ethics board approval, a netnography design was conducted to explore the publicly available donation profiles posted on GoFundMe^®^ to support individuals or families affected by OI. There are six overlapping steps of a netnography design [18], which include *Planning, Entrée, Data Collection, Interpretation, Ethics,* and *Research Representation*, which are delineated below.

*Planning* entails exploring the phenomenon of interest and the varying perspectives and meanings held by participants. This step includes exploring what content is being posted on various social media platforms and determining the foci of research, usually referred to as study questions [18]. The foci consisted of: (1) describing the GoFundMe^®^ Webpage profiles created for individuals with OI; (2) describing the reasons for requested funds and delineating the cost categories and items; (3) comparing funding goals with received funding; and (4) determining what factors may contribute to selecting and achieving funding goals and if specific items or requests are more likely to receive funding.

**Figure 1:**
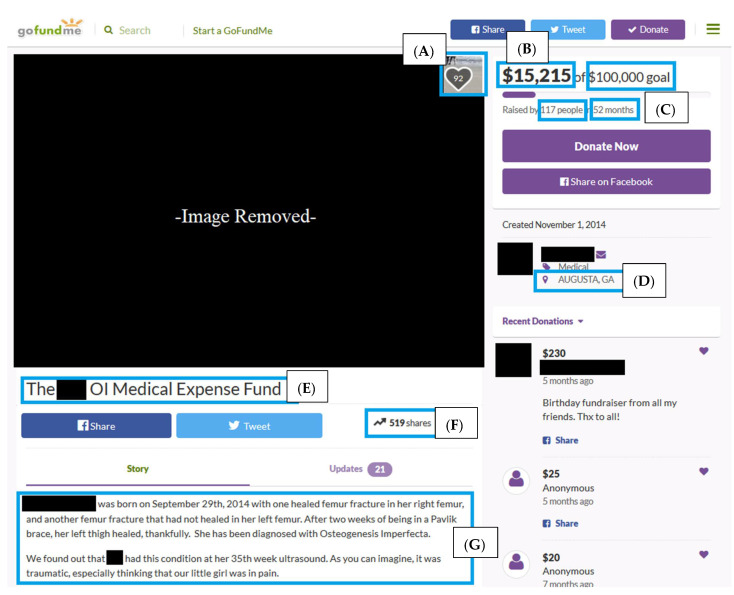
Sample of a GoFundMe^®^ Webpage Anonymously Displayed. (**A**) = number of “likes” by viewers; (**B**) = amount of money raised out of the total funding goal; (**C**) = number of people that have donated over a period of time; (**D**) = location of the profile creator; (**E**) = title of the webpage; (**F**) = number of times the webpage has been shared by other users/viewers; (**G**) = backstory behind the creation of the webpage.

*Entrée* involves choosing an appropriate online field. GoFundMe^®^ was chosen as an appropriate online field because a preliminary search revealed hundreds of profiles pertaining to individuals and families with OI. Along with its popularity, GoFundMe^®^ offered rich, detailed profiles containing quantitative and qualitative data, fulfilling the entrée recommendations of being relevant, active, interactive, substantial, heterogeneous, and data rich [18].The webpages included qualitative data in the form of biographies and images, as well as quantitative data related to requested financial support, geographical location, number of donors, likes, and Facebook, Inc., shares (Figure 1).

**Figure 2:**
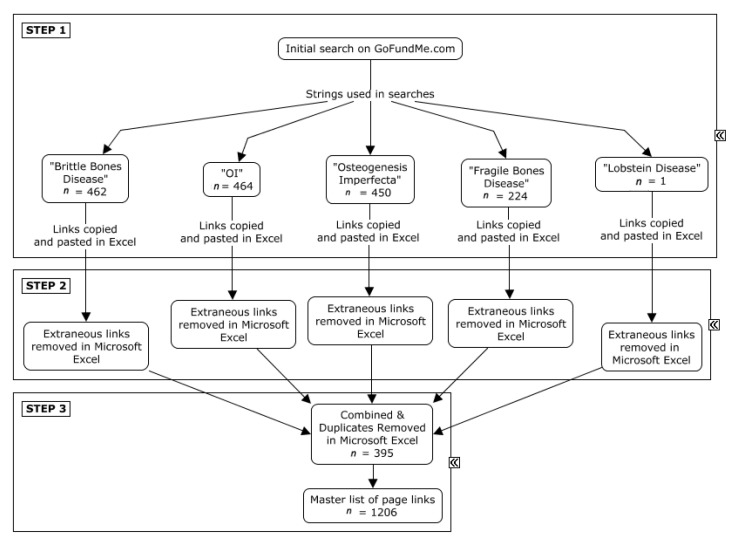
Flow diagram for Steps 1–3 of data collection method.

**Figure 3:**
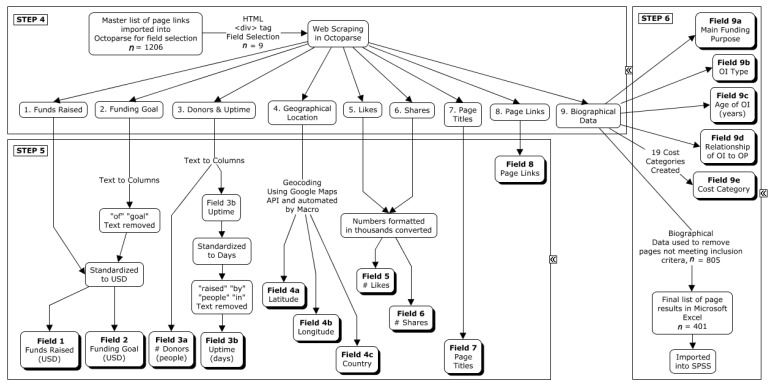
Flow diagram for Steps 4–6 of data collection method. # Donors (people) refers to the numbers of donors who donated to the GoFundMe^®^ Webpage profiles over the period of time.

*Data Collection* involves devising the necessary steps for data collection. These steps enable *web scraping*, also known as data mining. Web scraping is accomplished with software that extracts online text or media contained in webpages constructed using hypertext markup language (HTML). HTML is the standard language for webpages. All text and media elements for an individual page are displayed by tags located in the HTML code [23]. When data of interest are consistently displayed in identical locations across webpages of the same format, software may be instructed to search for the corresponding HTML tags and reliably extract the data [23]. The profiles included for analysis in IBM^®^ SPSS [24] were those intended to provide funding for an individual with OI and/or their family (e.g., for a child with OI). Six steps were used to facilitate data collection and prepare the dataset for analysis (Figure 2 and Figure 3).

*Step 1:* The Mozilla Firefox add-on Copy Links Advanced 1.3 [25] was used to generate five individual Microsoft Excel [26] spreadsheets, one for each string used to search: “brittle bones disease”, “OI”, “osteogenesis imperfecta”, “fragile bones disease”, and “lobstein disease” (Figure 2, Step 1).
*Step 2:* Extraneous links, which discussed legal information, common user questions, and other material unrelated to donation pages, were removed from the spreadsheets (Figure 2, Step 2).
*Step 3:* The five spreadsheets were merged into one master list (*n* = 1601 links), and 395 duplicate links were subsequently removed (Figure 2, Step 3).
*Step 4:* The master spreadsheet was imported into Octoparse [27], and web scraping was performed on 1206 pages to obtain the nine datasets listed in Figure 3, Step 4.
*Step 5:* The dataset was cleaned prior to statistical analysis. MapMakerApp.com, 1 May 2022 [28], which uses the Google Maps Application Program Interface (API), was used to provide a geocoded location based on the civic addresses of individuals with OI. MacroRecorder [29] was used to automate the process of searching for the GPS coordinates (Figure 3, Step 5).
*Step 6:* After combining the search results and removing duplicates, all biographical data were screened for eligibility. Included webpages were active donation pages on GoFundMe^®^ posted in English, French, or Spanish that were raising funds for an individual diagnosed with OI. Webpages were excluded if there were no indications that the person for whom the webpage was created had OI, or if the funds were intended for the family of a person with OI rather than the person who has OI. Excluded webpages also included those referring to “OI” as British/Australian slang for “Hi”, “Hi” in Portuguese, and/or describing individuals with “brittle bones” due to other medical conditions (e.g., side effects of chemotherapy). The content of included biographies was extrapolated to create Fields 9a, 9b, 9c, and 9d, corresponding with the main funding purpose, the OI type, the age of the individual with OI (child was less than 18 years), and the relationship of the individual with OI to the donation page creator, respectively (Figure 3, Step 6). Field 9e contained a total of 19 different cost categories.

*Interpretation* entails the steps for analyzing and interpreting the data. The *Cost of Illness Framework* guided the cost analysis. Cost of illness (COI) studies classified costs in three categories: direct, indirect, and intangible [9,12,30]. Direct costs can be healthcare or non-healthcare related, and include out-of-pocket expenditures, such as hospitalization, rehabilitation, and transportation costs. Indirect costs refer to productivity losses attributed to morbidity or mortality, and comprise informal care duties. Intangible costs, also known as psychosocial costs, encompass pain and suffering, usually measured in quality-of-life measures. Costs were deductively extracted from biographies and analyzed using other direct and indirect cost categories identified in pediatric COI studies [9,12,31,32]. Recognizing there may be other costs categories unique to OI, costs were also inductively identified and analyzed [9]. This approach was used to help foster a comparison of costs across other childhood conditions. Descriptive statistics were used to describe the webpage profiles and analyze each cost category (expressed in 2018 USD), followed by linear regressions of social media elements and Kruskal–Wallis analysis of sociodemographics, the funding goal, and funding received. Statistical analysis was performed using IBM^®^ SPSS [24], with significance set at a *p*-value of 0.05.

*Research Representation.* In a netnographic approach, an attempt to describe the *human element* of online human interaction and technological interaction, social interaction, and experience must be made [18]. Human elements consist of indicators of individuals’ unique or shared cultures, backgrounds, contexts, beliefs, values, personality, and other factors that affect how a person perceives and interacts with the social world. For example, shared language such as slang can be considered a human element, helping to situate the location, time, and context in which an online interaction is occurring; offer insight into the meaning of what is being communicated; and reveal information about the writer’s personal values and beliefs. To describe these human elements, the discussion of the descriptive statistics and quantitative analysis was written to showcase the costs associated with OI.

## 3. Experimental Results

The novel web-based approach of netnography was used to examine publicly available data from the GoFundMe^®^ website. The biographical data of 1206 GoFundMe^®^ web profiles were reviewed for inclusion (Figure 2, Step 3). In total, 401 webpages met the inclusion criteria (Figure 3, Step 6). 

### 3.1. Description of the GoFundMe^®^ Web Profiles Created for Individuals with Osteogenesis Imperfecta

**Figure 4:**
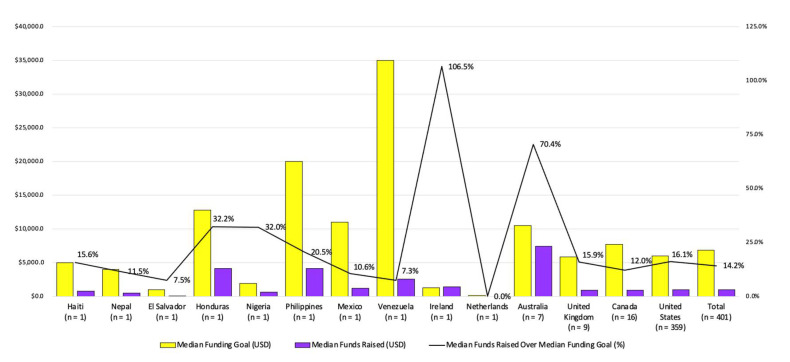
Descriptive Statistics of Donation Pages Organized by Country.

Of the 401 GoFundMe^®^ web profiles, 185 (46.1%) were created by family members, 119 (29.7%) were created by friends, and 97 (24.2%) were created by the individual with OI themselves. The individuals with OI were 48.4% male (*n* = 194) and 51.6% female (*n* = 207). Slightly fewer GoFundMe^®^ web profiles were created for adults with OI (*n* = 188, 46.9%) than for children with OI (*n* = 213, 53.1%). These web profiles were created in 14 countries, with the majority originating from the United States (*n* = 359, 89.5%) followed by Canada (*n* = 16, 4%), the United Kingdom (*n* = 9, 2.2%), and Australia (*n* = 7, 1.7%). The other 10 countries each had one profile (Figure 4).

### 3.2. Description of the 19 Cost Categories Requested for Funding by the Osteogenesis Imperfecta Community on GoFundMe^®^

In total, 19 different cost categories were requested for funding from the OI community. These cost categories consisted of direct healthcare (nine categories), direct non-healthcare (nine categories), and indirect (one category) (Table 1). Considering the percentage of cost categories cited per country, adapted vehicles (US 20.6%, Canada 18.8%), travel expenses (US 10.6%, Canada 18.8%), lost income (US 10.0%, Canada 18.8%), and mobility aids (US 8.4%, Canada 18.8%) were the most frequently requested. In the US, 8.9% of requests were also attributed to hospital admissions. The adapted vehicle cost category was ranked 16th in terms of funding success, despite having the largest median funding goal (USD 20,000) and being the most frequently requested OI cost category in the US (20.6%) and across all pages (19.7%).

### 3.3. Funding Goals against Received Funding

The median funding goal was USD 6812, with a median funding of USD 965 received (14.2% of the median funding goal) and median shortfall of USD 5847. There was a median of 14 donors, 14 likes, and 136 shares per page. The median uptime was 669 days. As shown in Table 2, the 37 web profiles that exceeded their funding goal (9.22% of total web profiles), with a median 760 days, had a median funding goal of USD 3500, with a median funding of USD 4285 received (122.4% of the median funding goal). For those that met their funding goal, there was a median of 57 donors, 57 likes, and 351 shares per page. Web profiles that reached their funding goal did not request donations for the following items: dental care, eye care, hearing care, legal fees, living expenses, medication, family planning, and physical therapy.

### 3.4. Contributing Factors to Selecting and Achieving Funding Goals and Specific Items or Requests That Increase Likelihood of Receiving Funding

Cost categories associated with the highest donation page funding goals were Adapted Vehicle (median request = USD 20,000), Medications (median request = USD 10,000) and Home Adaptions (median request = USD 10,000), as determined by Kruskal–Wallis. The main cost category requested influenced the amount requested (*p* = 0.011), with Adapted Vehicle, Medication, and Home Adaption having the largest effect, and Home Expenses, Recreational, and Eye Care having the least effect on the funding goal.

**Figure 5:**
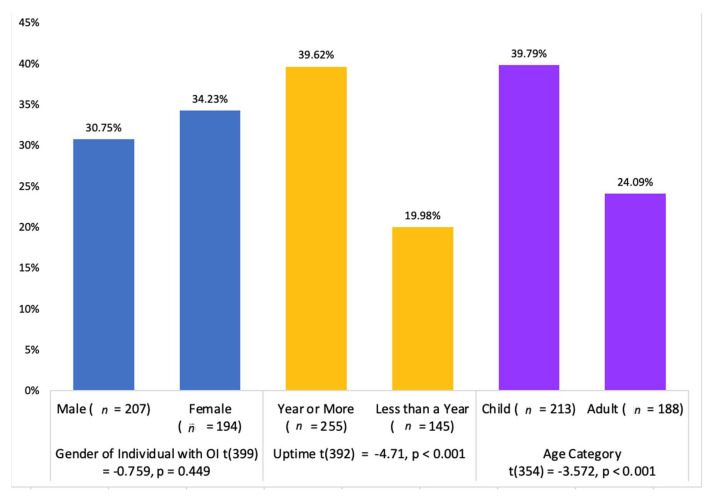
Comparing mean percentage of funding goal achieved for gender, uptime, and age.

Web profiles with uptime of over a year received an average of 39.6% of their funding goal, as opposed to 20.0% for web profiles posted for less than a year (*p* < 0.001). Web profiles created for children with OI received an average 39.8% of their funding goal, as opposed to 24.1% for adults (*p* < 0.001); children were 5.79% more likely to meet their funding goal. Since some web profiles may have received more funding partially due to a longer uptime, the success of each donation page was evaluated using the variable Percent Funding Goal reached per Month (PFPM). This metric served as a funding rate that controls for uptime. Web profiles created for children performed better in terms of PFPM (<0.001) compared to web profiles created for adults (Figure 5). The three cost categories with superior PFPM performance were Funeral Expenses, Mobility Aids, and Home Expenses, (*p* = 0.011), while Eye Care, Legal Fees, and Living Expenses had the least effect.

The log-transformed number of donors, likes, and shares was shown to significantly predict the log-transformed amount of funds raised by a GoFundMe^®^ web profile. A 1% increase in donors was associated with a 1.70% increase in funding received (*p* < 0.001); a 1% increase in likes was associated with a 1.74% increase in funding received (*p* < 0.001); and a 1% increase in shares was associated with a 1.18% increase in funding received (*p* < 0.001).

## 4. Discussion

The netnography offered unprecedented insight into the cost recovery efforts undertaken by the OI community on the website GoFundMe^®^ to help alleviate incurred economic changes, as well as characterization of the individuals for whom GoFundMe^®^ profiles were created. A total of 401 webpages from 14 countries for individuals with OI were analyzed. There was a fairly equal distribution of profiles across gender and age groups, with the majority of profiles originating in the US. Several known and new cost items and categories associated with the unique features of OI were identified. The analysis of funding goals and received funds allowed for a quantification of requested financial aid (median funding goal of USD 6812) and hierarchization of needs, permitting for an improved understanding of the support networks and success metrics at play.

The most frequently asked cost items from GoFundMe^®^ webpages included adapted vehicles (19.7%), travel expenses (11.2%), and lost income (9.7%). We also identified new cost categories which have not been previously described in OI literature, including legal fees, home expenses, living expenses, funeral expenses, and eye care [10]. Unlike funding requests with high PFPM (e.g., medication, funeral expenses), legal fees, living expenses, and eye care, which make up half of the newly identified cost categories, were also the least likely to attract funding. Legal fees are not captured in most pediatric COI literature [33,34,35], but may constitute a harmful and idiosyncratic part of the OI healthcare experience that differentiate OI from other childhood chronic illnesses. Families may accumulate legal fees due to child abuse allegations made prior to a diagnosis of OI [36,37], such as when a child with undiagnosed OI is admitted to the hospital with multiple unexplained fractures [38]. While eye care is not immediately associated with OI, OI can cause corneal thinning and sclera fragility, making the eyes vulnerable to trauma and progressive vision loss, requiring regular eye care [39]. Finally, living expenses (e.g., grocery costs, electricity bills) are rarely accounted for in COI studies [9,12], but may be a significant source of financial strain for the OI community due to additional expenditures such as home adaptations, treatment, lost income, and unexpected hospitalizations [4]. Legal fees, living expenses, and eye care are not readily associated with OI for individuals who are not familiar with the condition.

The cost categories with the highest funding goals include Adapted Vehicles, Home Adaptations, and Medications, which align with the high costs of disability and chronic illness [40]. For example, OI medication/treatments are typically lifelong, recurring expenses (e.g., bisphosphonate treatment) [41], which may be costly for families or individuals who are not or only partially covered by insurance. Furthermore, the cost of a new adapted vehicle can range from USD 20,000 to 80,000, while the cost of adapting a home can range from USD 800 to 75,000 [42] depending on the scope of the renovations (e.g., wheelchair ramps, elevator, hallway widening). However, home adaptations to accommodate the use of wheelchairs and other adaptive equipment may be a defining feature of OI, as well as other diseases requiring the use of mobility aids [43]. In contrast, adaptive equipment was minimally reported in a recent systematic review of the cost-of-illness evidence on ten rare diseases by Angelis et al. [44] which notably included Cystic Fibrosis, Hemophilia, and Fragile X Syndrome. Only Duchenne Muscular Dystrophy, a degenerative neuromuscular disease leading to loss of mobility, accounted for 63–83% of direct costs as stemming from equipment, devices, and aids [44]. Although financial support for adapted vehicles was the most common request, it ranked 16 out of 19 in terms of funding success, suggesting that adapted vehicles may not be perceived as medically urgent by donors. Nonetheless, the prevalence of requests for adapted vehicles aligns with the value of independence, autonomy, and mobility for people with disabilities [45,46,47], which can influence their quality of life and psychosocial wellbeing [48]. In addition, researchers have shown that travel costs such as car adaptation and acquisition remain costly for other childhood illnesses, such as cancer [32]. For individuals with OI, difficulties accessing an adapted vehicle can contribute to dependence on public transit and other individuals which may not be reliable, accessible, or available; this can limit their ability to participate in society, maintain desired employment, access far-from-home services, and participate in social/leisure activities [48,49].

A key aspect of funding success was receiving support from a well-developed social network. Social networks have been shown to have a direct positive effect on the provision of instrumental social support, including financial aid in previous COI studies [50,51] as well as in crowdfunding literature [15]. The importance of a family support network was highlighted in this study, and exemplified by the number profiles created, “liked”, shared, and funded by the social support system of individuals with OI or their families. The capacity to virtually reach out to social networks subsequently created website traffic which propelled the success of the campaign, such as through the use of “likes” and “shares”. Interestingly, funeral expenses, mobility aids, and home expenses received the most median funding. Greater donations were directed at children, especially for their funeral expenses and mobility aids, suggesting that empathy plays a strong role in the motivation to donate. Other have reported similar findings, noting that GoFundMe^®^ web profiles for children experience higher campaign success rates [15]. While pictures were not analyzed, empathy resulting from photographs depicting sad children may be more likely to receive donations due to a phenomenon described as *emotional contagion* [52]. Similarly, mention of funeral costs in the web profiles of children with mobility aids was likely to evoke empathic concern and feelings of distress in others; this form of “negative appeal” has been shown to be a powerful factor in eliciting donations [53].

The majority of donation pages originated in the US (*n* = 359, 89.5%), which may reflect the fact that most of the GoFundMe^®^ userbase is from the US (66.42%) [17]. As a result, most requests are derived from individuals navigating a non-single-payer nationwide system relying on public or private health insurance derived from for profit or non-profit insurers [54]. While studies in the US showcase the high costs of having a chronic illness or a chronically ill child [9,10,11], the webpages from Canada, Australia, and the United Kingdom suggest that financial stress caused by OI still exists in high-income countries with publicly funded health care systems. The shortcomings of healthcare systems may be perpetuated by gaps in healthcare resources, lack of comprehensive insurance coverage, poor access to or lack of government support, and indirect costs which are rarely addressed by health care systems (e.g., lost income, living expenses [40,55]). The U.S. Department of Health and Human Services acknowledges these shortcoming by suggesting crowdfunding as an option for families dealing with financial distress caused by OI [56]. With crowdfunding platforms’ reliance on popularity metrics, their propensity to accentuate social inequalities, and their inherent loss of confidentiality [57], researchers are questioning the ethical implications of such a practice [57,58]. These findings are consistent with research that identifies crowdfunding as a means to address the failings of formal safety nets [57,59]. Furthermore, the majority of GoFundMe^®^ pages were derived from high-income countries where there is widespread access to the internet [60], an essential prerequisite. Internet access in 2017 for Africa, Arab States, and the Asia/Pacific region were estimated to be 21.8%, 43.7%, and 43.9%, respectively, compared to 65.9% and 79.6% in the Americas and Europe [60]. A paucity of cost-of-illness OI research has been conducted in these countries, making it hard to assess the extent of this limiting factor; however, preliminary research suggests they are bearing financial costs as well [10].

### 4.1. Strengths and Limitations

This study uses a novel web-based approach to identify and monetize the costs associated with OI, which may contribute to financial distress. However, only one crowdfunding domain was used, and these profiles consisted of people who were willing to share their profiles for public, global viewing, and share their financial vulnerabilities. Analysis of other crowdfunding domains would be needed to assess the generalizability of these findings. The dataset represented a cross-sectional snapshot of OI-related GoFundMe^®^ activities, and included only data shared by the webpage creators. Biographies often lacked certain demographic details needed to fully characterize the sample of profiles. For example, few pages identified the OI type that the individual was diagnosed with. The financial data and geographical locations extracted from profiles were highly skewed, which limited the analysis and required data transformation and non-parametric statistics in some cases.

### 4.2. Implications

This study contributes to the literature describing the costs associated with OI and underscores the financial difficulties experienced by some members of the global OI community. Future work may benefit from sub-analysis of costly categories and the biographical narratives found on GoFundMe^®^ pages, permitting a more in-depth ethical interpretation of these costs [17] and appreciation of the neglected “psychosocial costs”. The rich qualitative data may lend insight to the situations of financial stress, potential for bankruptcy, and detailed issues with insurance and private health care. Additional research efforts are needed to identify the sources and magnitude of the financial difficulty to foster creative and effective solutions. As healthcare professionals strive to become more involved in policymaking, their understanding of costs must improve to optimize health care services. This study also contributes to the growing body of work that uses netnography to guide healthcare research, and hopefully encourages others to explore the informative, vast amount of publicly available data on the internet.

## 5. Conclusions

A novel web-based approach was used to describe varying costs associated with OI, showcasing the financial impacts individuals and/or families sought to alleviate through the world’s largest crowdfunding platform. The extraction of profile pages led to a characterization of GoFundMe^®^ OI users and underlined the support networks at play. The analysis of funding goals and received funds resulted in monetization of the requested financial aid, as well as a hierarchization of needs. These findings reinforce the need for further research to better understand associated costs and to provide optimal economic support for individuals living with OI and their families.

## Figures and Tables

**Table 1:** Summary of the 401 GoFundMe^®^ Web profiles Created to Raise Funds to Cover 19 Cost Categories Associated with Osteogenesis Imperfecta.

Cost Category	Cost Description	Median Funds Raised (USD)	Median Funding Goal (USD)	Funds Raised Over Funding Goal (%)	# of Donors	# of Uptime (Days)
	**Direct Costs**					
	*Direct Healthcare Costs*					
Dental Care (*n* = 10)	Any dental care costs incurred as a result of OI	1580	7250	21.8%	19.00	608
2. Eye Care (*n* = 3)	Eye care or surgery related to OI	110	1000	11.0%	2.00	304
3. Hearing Care (*n* = 1)	Audiology services or American Sign Language lessons that are needed as a result of OI	1120	7000	16.0%	18.00	7301
4. Hospital Admission (*n* = 33)	Costs associated with a hospital stay (imaging, laboratory tests, registration)	1530	10,000	15.3%	27.00	821
5. Surgical Intervention (*n* = 21)	Surgeries for fracture repairs, surgeries to prevent or palliate future fractures	827	10,000	8.3%	11.00	700
6. Medication (*n* = 5)	Difficulty with affording medications such as Forteo or Pamidronate	900	10,000	9.0%	10.00	274
7. Mobility Aids (*n* = 36)	Costs of repairing or obtaining wheelchairs, scooters, portable oxygen, walkers	1427	5000	28.5%	22.00	791
8. Physical Therapy (*n* = 16)	Costs associated with physical therapy equipment and services	2574	9000	28.6%	22.50	1034
9. Family Planning (*n* = 8)	Support for personal life elements such as in vitro fertilization, genetic counseling	878	7000	12.5%	18.00	1049
	*Direct Non-healthcare Costs*					
10. Adapted Vehicle (*n* = 79)	Modifying, obtaining, or repairing a vehicle that is wheelchair adapted	1135	20,000	5.7%	13.00	669
11. Home Adaptations (*n* = 21)	Home renovations such as lifts, ramps, kitchen adaptations, bathroom adaptations, or any attempt to improve wheelchair mobility within the home	800	10,000	8.0%	15.00	578
12. Home Expenses (*n* = 20)	Household bills, rent, utilities, furniture	467	3500	13.3%	4.00	319
13. Legal Fees (*n* = 4)	Legal expenses due to OI	13	5500	0.2%	0.50	380
14. Living Expenses (*n* = 3)	Food, groceries, clothing	440	3000	14.7%	6.00	335
15. Professional Development (*n* = 18)	Adaptive software, computer, education costs, travel for work/internship, adaptive devices, support for professional sports	648	5500	11.8%	9.00	639
16. Travel Expenses (*n* = 45)	The cost of traveling for health care services, including lodging, plane tickets, gas, ambulances, moving expenses	780	5000	15.6%	13.00	669
17. Recreational Activities (*n* = 20)	The need for funding for family vacations, holiday presents, concerts	353	3750	9.4%	10.00	669
18. Funeral Expenses (*n* = 19)	Funeral costs associated with relatives who have passed on as a result of OI	975	30,000	32.5%	25.00	760
	**Indirect Costs**					
19. Lost Income (*n* = 39)	Lost wages as a result of OI or caring for someone with OI	14,500	8000	18.1%	16.00	608
Total (*n* = 401)		965	6812	14.2%	14.00	669

Uptime: The number of days the GoFundMe^®^ web profile was publicly posted for funding.

**Table 2:** Summary of the 37 GoFundMe^®^ Web profiles whose Campaign Exceeded the Funding Goal Raising Funds for 11 of the 19 Cost Categories Associated with Osteogenesis Imperfecta.

Cost Category	Cost Description	Median Funds Raised (USD)	Median Funding Goal (USD)	Funds Raised Over Funding Goal (%)	Median # of Donors	Median # of Uptime (Days)
	**Direct Costs**					
	*Direct Healthcare Costs*					
Hospital Admission (*n* = 5)	Costs associated with a hospital stay (imaging, laboratory tests, registration)	9175	5000	183.5%	33.00	121.67
2. Surgical Intervention (*n* = 3)	Surgeries for fracture repairs, surgeries to prevent or palliate future fractures	3100	3000	103.3%	57.00	699.59
3. Mobility Aids (*n* = 8)	Costs of repairing or obtaining wheelchairs, scooters, portable oxygen, walkers	5777	5306	108.9%	96.00	958.14
	*Direct Non-healthcare Costs*					
4. Adapted Vehicle (*n* = 3)	Modifying, obtaining, or repairing a vehicle that is wheelchair adapted	5000	500	100.0%	13.00	851.68
5. Home Adaptations (*n* = 2)	Home renovations such as lifts, ramps, kitchen adaptations, bathroom adaptations, or any attempt to improve wheelchair mobility within the home	7560	6000	126.0%	100.00	912.51
6. Home Expenses (*n* = 2)	Household bills, rent, utilities, furniture	3895	2700	144.3%	44.00	577.92
7. Professional Development (*n* = 3)	Adaptive software, computer, education costs, travel for work/internship, adaptive devices, support for professional sports	7535	5500	137.0%	157.00	912.51
8. Travel Expenses (*n* = 3)	The cost of traveling for health care services, including lodging, plane tickets, gas, ambulances, moving expenses	9670	4000	241.8%	147.00	425.84
9. Recreational Activities (*n* = 2)	The need for funding for family vacations, holiday presents, concerts	838	800	104.7%	16.50	486.67
10. Funeral Expenses (*n* = 3)	Funeral costs associated with relatives who have passed on as a result of OI	4285	4000	107.1%	46.00	790.84
	**Indirect Costs**					
11. Lost Income (*n* = 3)	Lost wages as a result of OI or caring for someone with OI	2125	2000	106.3%	16.00	760.43
Total (*n* = 37)		4285	3500	122.4%	57.00	760.43

GoFundMe^®^ Web profiles that reached their funding goal did not request donations for the following items: dental care, eye care, hearing care, legal fees, living expenses, medication, family planning, and physical therapy. Uptime: The number of days the GoFundMe^®^ web profile was publicly posted for funding.

## Data Availability

For inquiries, please contact the corresponding author.
